# The Gender Difference in Depression: Are Elderly Women at Greater Risk for Depression Than Elderly Men?

**DOI:** 10.3390/geriatrics2040035

**Published:** 2017-11-15

**Authors:** Joan S. Girgus, Kaite Yang, Christine V. Ferri

**Affiliations:** 1 Department of Psychology, Princeton University, Princeton, NJ 08540, USA; girgus@princeton.edu; 2 School of Social and Behavioral Sciences, Stockton University, 101 Vera King Farris Drive, Galloway, NJ 08205, USA; christine.ferri@stockton.edu

**Keywords:** depression, gender differences, sex differences, aging, older adults

## Abstract

Numerous epidemiological reports have found that adolescent, young adult, and middle-aged adult girls and women are more likely to be diagnosed with unipolar depression and report greater symptoms of depression when compared to boys and men of similar ages. What is less well-known is whether this gender difference persists into late life. This literature review examines whether the well-known gender difference in unipolar depression continues into old age, and, if it does, whether the variables that are known to contribute to the gender difference in unipolar depression from adolescence through adulthood continue to contribute to the gender difference in the elderly, and/or whether there are new variables that arise in old age and contribute to the gender difference in the elderly. In this review of 85 empirical studies from every continent except for Antarctica, we find substantial support for the gender difference in depression in individuals who are 60 and older. More research is necessary to determine which factors are the strongest predictors of the gender difference in depression in late life, and particularly whether the factors that seem to be responsible for the gender difference in depression in earlier life stages continue to predict the gender difference in the elderly, and/or whether new factors come into play in late life. Longitudinal research, meta-analyses, and model-based investigations of predictors of the gender difference in depression are needed to provide insights into how and why the gender difference in depression persists in older age.

## 1. Introduction

It is well-known that females are more likely to be depressed than males, both in terms of unipolar depressive episodes and in terms of depressive symptoms [1,2]. However, while there is a lot of data on this for some age groups, there is much less data for others. For example, it is well-established that there is no gender difference in depression before early adolescence [3], the gender difference in depression emerges between ages of 11 and 15 [4], and that the size of the gender difference increases into adulthood when it apparently levels off [5]. For many years, the accepted wisdom was that the gender difference in depression became smaller and possibly even disappeared altogether in older adults, although there was a general acknowledgement that there was very little data [6]. Since there has been considerable research on depression in older adults in recent years, this seemed to be a good opportunity to review the literature to see the picture of the gender difference in depression in older adults that emerges. 

So far, a thorough discussion of the gender difference in unipolar depression in older adults is largely missing from major reviews of the gender difference in depression. There are several reasons for this. First, reviews of the gender difference in depression tend to focus on the emergence of the gender difference during adolescence and subsequently focus more on empirical studies of adolescent, college, and middle adult groups (e.g., [1,2,7]). When older adults are included in mixed samples, analysis of gender differences may not be stratified according to age or the sample size of older adults may be too small (e.g., [5]). This results in a limited ability to analyze or interpret data on the gender difference in unipolar depression in older adults in mixed samples. Second, some have expressed uncertainty over whether the gender difference in depression exists in adults aged 55 and older (e.g., [4,8]). Some uncertainty may come from theorizing and data showing that overall rates of depression and negative affect are attenuated in older cohorts (e.g., [9,10]), although a decline in the prevalence of depression in older age does not necessarily mean that the gender gap will narrow. Because of the relative absence of studies with data on individuals between the ages of 60 and the end of the life span, the earlier reviews necessarily focused on studies with data from middle-aged and young-old adults, rather than on an elder population. 

In this paper, we will review what is known about the gender difference in unipolar depression in old age (age 60 and older). The primary question we will try to answer is whether there is sufficient empirical data to support the existence of a gender difference in depression in older adults. If the gender difference in depression does exist in older adults, we will examine whether the major psychosocial variables that are thought to be responsible, at least in part, for the gender difference in depression in teenagers, young adults, and middle-aged adults play a similar role in depression in older adults, and/or whether there are new psychosocial variables that are responsible for a gender difference in depression in older adults. Finally, we will try to specify the most important research questions that remain to be answered in order to develop a reasonably complete picture of the gender difference in depression throughout the life course.

## 2. Epidemiology of the Gender Difference in Depression in Older Adults

The first goal was to answer the following question: Is there a gender difference in depression in older adults who are at least 60 years old? We conducted a literature search in March 2017, using the Academic Search Complete, PsycINFO, and MedLine (ProQuest) databases. For each database, the search terms “gender difference”, AND “depression”, AND “elderly or aged or older or elder or geriatric” were used. This returned 713 abstracts from Academic Search Complete, 3788 abstracts from PsycINFO, and 3155 abstracts from MedLine. Full articles were retrieved and included in our review if they met the following inclusion criteria: empirical research articles published in peer-reviewed journals; participants aged 60 or aged 50 and above with the mean age of the participants at least age 70; both women and men as participants; and, a report of unipolar depression by gender. Lifespan studies were only included in our review if depression data was reported by gender for the group of participants who were 60 and above. Duplicate abstracts were consolidated. These criteria were met by 85 articles that were published between 1980 and 2017. In these 85 articles, the most commonly used measures of depression were the well-validated Center for Epidemiologic Studies Depression scale (CES-D) [11], which was used in 36 (42%) of the studies, and the Geriatric Depression Scale (GDS) [12] which was used in 19 (22%) of the studies. One study used both CES-D and GDS scales [13]. The CES-D and GDS are valid instruments for the assessment of depressive symptoms in geriatric samples [12,14,15] The other 29 studies used 24 different measures, including structured clinical interviews, physician diagnoses, and other self-report measures of depressive symptoms (e.g., [16,17,18,19]). These 85 studies included participants from 34 different countries, and from every continent except for Antarctica. The vast majority of the articles (72%) were published in the last 10 years, reflecting the increased interest in the subject of depression in the elderly in recent years. 

In 69 (81%) of the 85 studies, older women, as compared to older men, had a significantly greater likelihood of a depression diagnosis based on clinical interviews or cut-off scores, or significantly more depressive symptoms on standard measures. Of the 69 studies, four found a significant gender difference in some groups but not others. In 14 of the 85 studies (16%) there was no significant difference in depression scores or diagnoses between women and men. In two of the 85 studies (2%), men had significantly greater depression scores than women. Taken altogether, this answers the central question of this paper: the gender difference in depression, with women more likely to be depressed than men, is found in people over the age of 60. (See Table S1 in Supplemental Materials for the full list of articles reviewed, e.g., [20,21,22,23,24,25,26,27,28,29,30,31,32,33,34,35,36,37,38,39,40,41,42,43,44,45,46,47,48,49,50,51,52,53,54,55,56,57,58,59,60,61,62,63,64,65,66,67,68,69,70]).

Only 16 (18%) of the 85 studies that met the criteria for inclusion in this review did not find a significant gender difference in depression, in which women scored higher than men. In these 16 studies, the characteristics of the samples and the ways in which depression was assessed did not seem to differ in any significant way from the 69 studies in which a gender difference in depression was found. Furthermore, these 16 studies reported data on samples from 11 different countries (France, Italy, Sweden, Taiwan, United States, Australia, Germany, Brazil, Singapore, China, South Korea).

In summary, 69 of 85 (81%) studies that reported on the gender difference in depression in the elderly found that older women were more likely to have a diagnosis of depression or a greater number of depressive symptoms when compared to older men. Although interpretation of the epidemiological findings is made somewhat more difficult because only a comparatively small percentage (29%) of the studies excluded participants based on conditions such as dementia, cognitive function, chronic illness, disability, and co-morbid psychological disorders, the consistency of the basic finding of a gender difference in depression in the elderly extends the consistent epidemiological finding that there is a gender difference in depression among adolescents, younger adults, and middle-aged adults (e.g., [1,71]). 

## 3. Gender Differences in Depression across the Lifespan

Some empirical studies have asked whether the gender difference in depression changes across development stages from adolescence and young adulthood into late life. Our screening process returned 10 studies with participants from 18 different countries that addressed the question of whether the gender difference in depression changes in magnitude across the life span. The results of these analyses were mixed. Three of the studies reported that the gender difference in depression, while remaining significant, was narrower in elderly adults as compared with young- and middle-aged adults [72,73,74]. Three studies reported that the size of the gender gap in depression increased with age [75,76,77]. Finally, four studies found that the gender difference in depression remained the same across age groups [78,79,80,81]. 

One possible reason for the mixed results is the differences in the age groups that were analyzed in the various studies. In the studies that found no change in the gender difference in depression over age, one compared all of the participants under age 55 with all of the participants over age 55 [79], a second compared all of the participants under age 50 with five, five-year cohorts over the age 50 [80], and a third compared of the participants over age 55, divided into eight, five-year, cohorts [78]. In other words, in these three papers, most of the data were drawn from participants over the age of 50. The fourth study that found no difference in the gender difference with age compared a small group of 18-29 year olds to a small group of 50–76 year olds [81]. In the studies that found a decrease in the gender difference in depression with age, the participants were spread more completely throughout the life span. One compared a sample of 20–35 year-olds to a sample of 70–85 year-olds [73]; a second compared samples of three age groups, ages 25–35, 45–55, and 65–75 [72]; and, the third compared a slightly different set of three age groups, ages 20–24, 40–44, and 60–64 [74]. Of the three studies that found an increase in the gender difference in depression with age, one had participants who ranged in age from 45 to 85, divided into four, 10-year cohorts [77], one had participants who ranged in age from younger than 30 to 80 and older, divided into seven cohorts [76], and the third had participants divided into 15-year cohorts, beginning with age 18 and concluding with a cohort of over 65 year-olds [75]. 

In general, the studies that explored the possibility of changes in the gender difference in depression across the life span used a cross-sectional approach, with different participants at different ages. As a result, cohort effects cannot be ruled out. Large-scale epidemiological studies have revealed that a cohort effect is present for depression, with more recent cohorts reporting higher rates of depression when compared with older cohorts [9,10,82]. It is not clear, however, that higher rates of depression in more recent cohorts would necessarily affect the size of the gender difference in depression. Three of the studies combined longitudinal data gathering with a cross-sectional design [74,76,80]. Nguyen & Zonderman (2006) and Mirowsky (1996) followed their participants over a 6–10 year period, and Leach et al. (2008) followed their participants over a 20-year period. One of these studies found an increase in the gender difference in depression with age [76]; one of these studies found a decrease in the gender difference in depression with age [74]; and, the third study found no change in the size of the gender difference in depression with age [80]. The particular age cohorts analyzed differed substantially among these three studies and so the lengths of time and the ages included in them may not have been sufficient to test the question of whether there are changes in the gender difference in depression as people enter and move through old age.

All in all, the currently available data are clearly mixed on whether and how the gender difference in depression changes across the life span. There is no readily perceivable pattern based on the sampling procedures (or the depression measure) used in those studies that have tried to examine this question. 

Two studies examined the incidence of the initial depressive episode between the ages of 18 and 65, and asked whether more women than men experienced an initial depressive episode at each age [83,84]. In both of the studies, the probability of an initial episode of depression was higher for women than for men across this age range. However, the probability of an initial episode of depression declined with age for both women and men, with the gap between them narrowing as the probability became extremely low for both genders in old age. On the other hand, in a study of initial depressive episodes in a longitudinal sample that was tested every five years between the ages of 70 and 85, Palsson, Ostling, and Skoog (2001) found that initial depressive episodes increased across this age range for both men and women, and women were consistently more likely than men to experience an initial episode [85]. 

## 4. Psychosocial Predictors of the Gender Difference in Depression

While the psychosocial predictors of depression have been extensively studied in people of all ages, the psychosocial predictors of the gender difference in depression have been studied much less, and almost not at all in older adults. What research there is on the psychosocial predictors of the gender difference in depression has generally focused on adolescents and young adults, and utilized diathesis-stress models. In this research, the diatheses are vulnerabilities that make individuals more susceptible to depression when stressors occur in their lives, and the question is which stressors and which vulnerabilities are more prevalent in the lives of females than in the lives of males [86]. The research on the psychosocial predictors of the gender difference in depression in older adults, however, has generally not utilized any underlying model, but instead has usually looked at single variables that might be related to the gender difference in depression. While sometimes multiple variables are included in a single study, the question being asked is almost always which of these variables accounts for the most variance in the gender difference in older adults. 

In addition, many researchers have looked separately at which variables predict depression in older women and which variables predict depression in older men. These studies rarely ask whether there are gender differences in the psychosocial variables that predict depression in each gender (and whether any gender differences in those variables might lead to women being more negatively affected than men). There are very few studies that look at whether there is a gender difference in a predictor variable that predicts the gender difference in depression. This is unfortunate because only with such studies can we conclude that that variable can, at least in part, explain the gender difference in depression in the elderly. 

In this section, we will examine the results of the studies that have looked at gender differences in psychosocial variables that might predict the gender difference in depression in older adults. First we will consider whether there is evidence that the variables that predict the gender difference in depression in adolescents and younger adults might also predict the gender difference in depression in older adults, and then we will consider whether there are additional variables that arise in old age that might be responsible at least in part for the gender difference in the elderly. 

This section combines studies that were done in many different countries. In some studies, data from several different countries were examined. The results were quite consistent across countries, and so the results are reported without considering the countries in which the data were gathered. 

## 5. Psychosocial Predictors of the Gender Difference in Depression That Occur throughout the Life Span

In adolescents and younger adults, the predictors of the gender difference in depression that have been identified include: stressors, coping styles (e.g., rumination); depressogenic personality styles (e.g., sociotropy); social support; the physiological changes of puberty; gender role intensification, sexual abuse and assault; and, negative body image [2,7,86]. The physiological changes of puberty and gender role intensification have been viewed as specific to adolescence and young adulthood, while negative body image and sexual abuse and assault have been seen as more prevalent in adolescence and young adulthood than in later life stages. Coping styles, depressogenic personality styles, and some stressors, on the other hand, would seem to be candidates for predictors of the gender difference in depression throughout the life span. In this section, we will explore whether these variables seem to predict the gender difference in depression in the elderly, as they do the gender difference in depression in adolescents and young adults.

### 5.1. Stressors/Negative Life Events

There are many different kinds of stressors. The most commonly measured stressors in the depression literature generally are negative life events that occurred within the previous year. However, very few studies have asked whether there are gender differences in negative life events in the elderly that are related to the gender difference in depression in this age group, despite the fact that negative life events occur frequently in the lives of the elderly. In the few studies that have examined this question, it is not clear whether older women experience more negative life events than older men: in two studies they did [87,88] and in two studies they did not [73,89]. Chan, Kwok et al. (2012) found that elderly women had significantly more depressive symptoms than elderly men, but did not analyze their data to see whether the gender difference in negative life events predicted the gender difference in depression [88]. Seematter-Bagnoud et al. (2010) did not analyze their data either for a gender difference in depression or for a possible relationship between the gender difference in negative life events and a possible gender difference in depression [87]. 

### 5.2. Coping Styles

Cognitive coping skills are commonly considered to be predictors of the gender difference in depression in adolescents and younger adults ([90], for meta-analysis, see [91]). The most extensively researched coping style that has been linked to the gender difference in depression is rumination, which was initially proposed by Susan Nolen-Hoeksema. Rumination occurs when stressors lead to negative moods and individuals respond to the negative moods by dwelling on them, their causes, and their implications, rather than engaging in either problem-solving or distraction [92]. Women ruminate more than men when faced with a stressor [93], and this gender difference in rumination is well established as a risk factor for the gender difference in depression in adolescents and adults [94]. Only a few studies have examined rumination in old age [72,73,74,81]. These studies all compared older adults with younger ones. Collectively, they found that rumination declines with age, but at all ages, women ruminate more than men. Those studies that examined whether rumination mediates the relationship between gender and depression found that it did [72,73,81]. 

There is a substantial history of dividing coping styles into approach styles (e.g., problem-solving) and avoidance styles (e.g., avoiding thinking about a problem) [95]. Two studies have examined gender differences in coping styles in the elderly [96,97]. Both found that elderly women use avoidance coping styles more than men do, and Lutzky and Knight (1994) found that this gender difference was related to the gender difference in depression [96]. In these two studies, women also used approach coping styles (especially seeking social support) more than men did, but only Lutzky and Knight (1994) tested whether this gender difference was related to the gender difference in depression, and they found that it was not [96]. 

### 5.3. Interpersonal Orientation (Sociotropy/Dependency)

Some depressogenic personality characteristics that are quite commonly assessed in younger samples have not been examined in older samples. For example, dependency and sociotropy are interpersonal orientations that have been proposed to contribute variance to the gender difference in depression in younger samples [98,99,100]. This review of the gender difference in depression did not find any articles that examined dependency and sociotropy as predictors of the gender difference in depression in the elderly. While we have some preliminary data that suggests that sociotropy declines with age [101], it is unclear whether the gender difference in sociotropy that is still present in old age explains some of the gender difference in depression in the elderly.

### 5.4. Social Support

On the face of it, social support looks like a potentially important variable when considering the predictors of depression. In general, social support is seen as buffering against depression; that is, it is assumed that social support protects a person against depression [102]. Insofar as social support is viewed as a buffering variable, the obvious hypothesis would be that it is the absence of social support that predicts depression. Therefore, if a gender difference in social support plays a role in the gender difference in depression in the elderly, elderly women would be expected to have less social support than elderly men. Rather, there is significant evidence that older women have higher rates of social contact, support, and participation, and more extensive social networks, than older men [89,103,104,105,106,107]. Some research shows similar quality and quantity of social relations in elderly men and women [108,109], and other studies have found that elderly men had higher perceived level and perceived availability of social support than elderly women [110,111]. Even within a single study, there are sometimes results that could be seen as contradictory. For example, van Grootheest et al. (1999) found that elderly women reported receiving significantly more emotional support and significantly less instrumental support than elderly men [105]. Leach et al. (2008) reported more positive support and fewer negative events from friends for elderly women as compared to elderly men, but also reported no difference between elderly women and men in positive support and negative events from family [74]. Lee & Lee (2011) reported that older women visited more frequently with friends than older men did while older men visited more frequently with their children than older women did [112].

One of the reasons for the mixed results may be that there are many different ways of defining social support, and it is difficult to figure out whether there is any comparability among them. For example, social support can be either instrumental or emotional. It can be defined as the size of a person’s social network or as whether a person has a best friend or confidante. It can be seen as coming from family or from friends. One or more of these kinds of social support could have more of an effect on one gender or the other, and these effects could be positive or negative. For example, it is possible that elderly women are more vulnerable to the absence of social support than elderly men and that this difference in vulnerability could account in part for the gender difference in depression.

## 6. Psychosocial Predictors of the Gender Difference That Are More Prevalent in Older Adults

The psychosocial variables that are more prevalent in old age than in younger age groups can best be characterized as new stressors that are more likely to arise or intensify in old age. These include: widowhood/living alone; illness; cognitive decline; financial strain/poverty; and, caregiving. Each of these variables increases in old age, and the question then becomes: are there gender differences in these variables in old age and, if so, do these gender differences predict the gender difference in depression in old age?

### 6.1. Widowhood/Living Alone

Marital status and living arrangements are the most studied psychosocial variables in the literature on depression in the elderly. These are not exactly the same variables although they certainly overlap; for example, it is possible for an elderly person who is widowed or divorced to be living alone or to be living as part of an extended family. We will first examine the literature on marital status, and then we will explore the literature on living arrangements. 

The data consistently show that elderly women are less likely to be married and more likely to be widowed than elderly men [13,74,78,87,89,105,107,113,114]. Although the literature makes it clear that elderly adults who are married have less depression than elderly adults who are widowed/divorced/separated [107,115,116,117,118], there is much less data on whether the gender difference in marital status is related to the gender difference in depression. Since women are more likely than men to be widowed/divorced/separated and since people who are widowed/divorced/separated have more depressive symptoms than people who are married, it would not be surprising if marital status were a predictor of the gender difference in depression, but direct tests of this have been infrequent, and have not supported this view. Rather, what little data there are suggest that women who are married are more depressed than men who are married, but there is not a gender difference in depression in older adults who are separated/divorced/widowed [74,105,114,117]. Some investigators have focused specifically on widowhood as a life event that predicts geriatric depression. In general, these studies found no gender differences in depressive symptoms after widowhood [111,118]. 

Other investigators have proposed that a gender difference in living alone might be a predictor of the gender difference in depression. Many more elderly women live alone than elderly men [13,87,116]. However, elderly men who live alone have more depressive symptoms than elderly women who live alone [116,119], exactly the opposite of what one would expect if a gender difference in living alone were a predictor of the gender difference in depression. 

### 6.2. Poor Health/Chronic Illness

Health status seems to be an obvious variable to examine in relation to the gender difference in depression in the elderly since health tends to decline as people age. Health can be measured in a number of ways: number of chronic conditions, ability to do the tasks of daily life, and self-ratings of health are the approaches that have been used most frequently in studies of the elderly. 

The first question, however, is whether there is a gender difference in health. Do elderly women have poorer health and more chronic conditions than elderly men? In general, the answer to this question seems to be yes. There is a gender difference in health status in the elderly, with women experiencing more chronic conditions, demonstrating less ability to perform daily tasks, and subjectively reporting poor health [13,74,78,87,106,112,120]. Given this, health status becomes a candidate for predicting the gender difference in depression in the elderly. Only one study has asked this question directly. Noh et al. (2016) found that there was no difference in depression scores between men and women who were not diagnosed as having a disability but women who were diagnosed as having at least one disability were significantly more depressed than men who were diagnosed as having at least one disability [120]. 

### 6.3. Cognitive Decline/Dementia

The studies of the relationship between cognitive decline/dementia and depression in the elderly differ from studies of the relationship between other variables and depression in the elderly in an important way. Whereas, studies of other variables generally ask whether the variable predicts depression, studies of cognitive decline/dementia ask whether depression predicts cognitive decline. While the reason(s) for this is not explicitly discussed in the literature, it seems likely that it reflects the difficulty of assessing depression in individuals with dementia. There are considerable data that older women are not only more likely to be depressed than older men, but also are more likely to be diagnosed with dementia, especially over age 85 [121,122,123,124], although some studies suggest that this gender difference is not found in North America and Australia [124,125]. Yet, despite the fact that elderly women seem more likely to be diagnosed both with dementia and with depression than elderly men, the data do not support the idea either that the gender difference in depression is related to the gender difference in dementia. 

The data on whether depression is a risk factor for or a correlate of cognitive decline are mixed, and gender differences have rarely been reported. In the studies reviewed by Sevick, Rolih, and Pahor (2000), the data on depression as a predictor or correlate of dementia or cognitive decline were mixed, with no gender differences being reported [126]. In the meta-analyses conducted by Jorm (2000), there appeared to be a small but significant association between a history of depression and subsequent dementia and cognitive decline over periods of less than a decade, but again no gender differences were reported [127]. In a study of individuals with dementia, elderly women with mild dementia had more depressive symptoms (on a scale completed by a caregiver) than elderly men with mild dementia, but there was no gender difference in caregiver rated depressive symptoms in individuals with moderate dementia [128]. Overall, then, it is not clear whether a history of depression is a risk factor for dementia and cognitive decline, and the data generally suggest that there are no gender differences in whatever relationship there is between depression and cognitive decline. 

### 6.4. Financial Strain/Poverty

Although financial strain and poverty are thought to be more prevalent in women than in men throughout the life span, perhaps surprisingly, we found very few studies in the depression literature that reported whether elderly women experience more financial strain and poverty than elderly men. Those studies that have examined whether elderly women are more likely to be poor than elderly men have found this to be the case [129,130]. Most of the studies that have asked whether financial strain or poverty predicts depression in the elderly have asked this question separately for men and women [130,131,132,133,134,135]. There do not seem to be any studies that have asked whether a gender difference in financial strain or poverty predicts the gender difference in depression in old age. 

### 6.5. Caregiving

Caregiving is by no means unique to older adulthood, but it is certainly a demand placed on many older adults. Pinquart and Sorenson (2006) conducted a meta-analysis that found higher rates of depressive symptoms in female caregivers than in male caregivers, with women reporting higher levels of burden, more hours of care, more caregiving tasks, and more personal care than male caregivers [108]. This meta-analysis combined spouses, children, and other caregivers so, although those needing care were all elderly, the caregivers were not. There were several other findings of interest: the gender difference in depression was larger for older caregivers as compared to younger, and for samples of caregivers as compared to samples that were not selected for caregiving. Not surprisingly, this meta-analysis found gender differences in several stressors that were specific to caregivers (e.g., hours of care per week), although, somewhat unexpectedly, the meta-analysis did not find any gender difference in either formal or informal support. After controlling for stressors and social support, the meta-analysis found that the gender difference in depression in caregivers was no longer larger than the gender difference in depression in a sample not selected for caregiving, supporting a stress and coping model of the additional gender difference in depression found in caregivers. 

The Pinquart and Sorenson (2006) meta-analysis examined studies in which those receiving care might be debilitated in various ways [108]. Other studies have focused on the caregivers of those with dementia or Alzheimer’s disease [110,136]. Although these studies did not directly test whether there were caregiver variables that predicted the gender difference in depression in the caregivers, they did find that female caregivers had higher subjective burden and perceived stress than male caregivers.

## 7. Conclusions

The present review found strong empirical evidence for the gender difference in depression among older adults aged 60 and above. Older women scored higher than older men on dimensional measures of depressive symptoms and older women have higher rates of diagnosis of unipolar depression compared to older men. This pattern was observed in 69 (81%) of 85 studies. The majority of studies included in this review assessed depression using dimensional self-report instruments of depressive symptoms. Although self-report instruments, such as the CES-D and GDS, are not the same as a diagnostic clinical interview, they nevertheless are valuable tools for depression screening and assessment of symptom severity, are highly predictive of well-being, and are valid assessments in older adults [12,14,15]. 

Although subclinical levels of depressive symptoms are more commonly experienced in geriatric samples than major depressive disorder [137], subclinical depressive symptoms contribute substantially to a decreased quality of life, including cognitive decline, longer hospital stays, and poorer health in late life [137]. Therefore, the substantial evidence for the existence of a gender difference in depression, even at subthreshold levels, is a consequential finding.

The literature reviewed for this paper was impressive in its geographic and cultural diversity, with every continent except for Antarctica being represented in the papers reviewed. The majority of the papers reported data from North America, Asia, and Europe, with fewer studies taking place in South America, Africa, and Australia. Population-based samples predominated in the studies reviewed, with remarkably little dependence on convenience samples. Taken altogether, the existence of a gender difference in depression in the elderly, with women being more likely to be depressed, appears to be a very robust finding. 

While the existence of the gender difference in depression in the elderly seems to be solidly established, we know much less about why this gender difference occurs in the elderly, the magnitude of this effect over ages, or whether this effect occurs in the elderly for the same reasons that it occurs at earlier life stages. In particular, we were unable to ascertain whether the data were similar for older adults (age 60–80) and for very old adults (older than age 80). Those studies that explored the gender difference in depression across a broader age range in order to examine whether the gender difference in depression changed in magnitude across the life span provided mixed data, perhaps because most of these studies used different age ranges [72,73,74,75,76,77,78,79,80,81]. However, even among those studies that included cohorts of participants ranging in age from the late teens to over 65, the results were mixed: three found an increase in the gender difference in depression in later cohorts [72,73,74], and three found a decrease in the gender difference in depression in later cohorts [75,76,77].

In general, the vast majority of the studies that explored predictors of depression in the elderly looked at what predicted depression in women and men separately. There were relatively few studies that reported gender differences in potential predictor variables, and an even smaller number of studies that actually tested whether the gender differences in predictor variables accounted for a significant part of the variance in the gender difference in depression. In addition, most of the studies focused on stressors that increase dramatically in old age, such as widowhood/living alone, poor health/chronic illness, cognitive decline, financial strain/poverty, and caregiving. There were relatively few studies with elderly participants that explored predictors, such as negative life events, coping styles, and interpersonal orientation, which have been extensively researched in adolescents and adults. The only predictor of the gender difference in depression that has been studied extensively throughout the life span is social support, but the data on the role of social support are very mixed and difficult to interpret, probably because there are so many different ways to define social support (and its absence).

There does seem to be some indication that dysfunctional coping styles, such as rumination and avoidance coping, are more prevalent in elderly women than in elderly men [72,73,74,81,96,97], just as they are in adolescents, young adults, and middle-aged adults ([72,138], for meta-analysis, see [94]). However, considerably more research will be needed in order to understand the role that these coping styles may play in the gender difference in depression in the elderly. Research is also needed on the question of whether interpersonal orientation (sociotropy/dependency) plays a role in the gender difference in depression in the elderly as it apparently does at younger ages [98,99,100].

Although particular stressors—widowhood/living alone, poor health/chronic illness, cognitive decline, financial strain/poverty, and caregiving—have been extensively studied in the elderly, the broader range of negative life events that have been studied in adolescents and adults, and that generally represent the stressors in diathesis-stress models of depression, are notably absent from the literature on gender differences in old age. More research on the broad range of negative events in the lives of the elderly might prove informative for understanding the gender difference in depression in this age group.

Although we know more about the particular negative events of old age than we do about negative life events in general, we do not know nearly enough about any of them to be able to specify whether a particular negative event makes a contribution to the gender difference in depression in the elderly. People who are not married (widowed, divorced, or separated) are more likely to be depressed than people who are married, and elderly women are much less likely to be married than elderly men [107,115,116,117,118]. However, direct tests of not being married as a contributor to the gender difference in depression are scarce, and the few studies that have looked at this have found that elderly women who are married are more depressed than elderly men who are married, but there is no difference in depression between women and men who are widowed, divorced, or separated [74,105,114,117]. In a major meta-analysis, Pinquart and Sorenson (2006) found clear evidence that female caregivers reported more depression than male caregivers [108]. However, the studies in this analysis included caregivers of a wide range of ages, while those receiving care were all elderly. Nor did this meta-analysis test whether being a caregiver of an elderly person accounted in part for the gender difference in depression in the caregivers. Clearly research that focuses on the elderly caregivers of elderly care recipients is needed before there will be a clear picture of the role that caregiving might play in the gender difference in depression in the elderly. Poor health and chronic illness, cognitive decline, and financial strain and poverty need even more research before they can be considered possible contributors to the gender difference in depression. What data there are suggest that elderly women suffer from poorer health in general than elderly men [13,74,78,87,106,112,120], but we know very little about whether poorer health is related to the gender difference in depression. Elderly women are apparently more likely to be poor than elderly men [129,130], but again the connection between this and the gender difference in depression is obscure. Gender differences in cognitive decline and dementia, and their possible relationship to the gender difference in depression, are even more difficult to examine, largely because it is so difficult to assess depression in individuals with dementia. As a result, most of the research on the question of the relationship between cognitive decline and depression has asked whether depression is a risk factor for cognitive decline rather than whether cognitive decline is a risk factor for depression [126,127,128].

It seems clear that there is a new set of stressors that arise in old age. Unfortunately, there is not sufficient research to be certain whether any of these new stressors contributes to the gender difference in depression in the elderly. There is, however, a hint in the literature that the risk factors for a depression diagnosis in the elderly may be different from the risk factors at younger ages. Studies have documented a U-shaped function for the gender difference in initial diagnosis of depression between the ages of 18 and 65, with essentially no gender difference at the youngest and oldest ages [83,84]. However, a study that examined the gender difference in the initial diagnosis of depression in a sample of individuals aged 70–85, found that initial depressive episodes increased for both women and men across this age range with women being consistently more likely than men to experience an initial episode [85]. This strongly suggests that a new set of risk factors, which are more prevalent in women than in men, enters the lives of individuals after age 65.

Severe and moderate (but not mild) depression is also associated with increased mortality in older adults [139,140], and chronic depression is associated with an increased mortality in the elderly [139]. The data on gender differences in the relationship between depression and mortality are mixed, however. Although elderly women are more likely to be depressed than elderly men, some studies found that men who are depressed have a higher risk of mortality than women who are depressed [139,141]. At least one other study, however, found an increased risk of mortality for depressed women but not for depressed men [142]. Older men are also at higher risk for death from suicide than older women, and the reasons for suicide in older adults also differ by gender [143]. 

### 7.1. Recommendations for Future Research and Clinical Application

We designed this review of the literature on the gender difference in depression in the elderly to examine, first, whether the well-known gender difference in depression among adolescents, young adults, and middle-aged adults, was also consistently found in the elderly and, second, to examine what was known about risk factors for depression in the elderly. The present review establishes the existence of the gender difference in depression among the elderly and reveals important gaps in the literature about what factors are responsible for this gender difference. Below, we describe ways to address these gaps through empirical research. We also briefly touch on applications of these findings for clinical practice, especially as the applications pertain to depression screening of senior populations.

### 7.2. Theory-Based Approaches to Understanding the Gender Difference in Depression

Although the general literature on the gender difference in depression, which is almost entirely based on samples of adolescents and young adults, has examined and compared various models designed to explain the gender difference [83], model-driven approaches to understanding the gender difference in depression in elderly adults are noticeably rare. In the literature on the etiology of the gender difference in depression in adolescence, three models have been proposed to explain why girls are more likely than boys to experience depression: the diathesis-stress model; the mediation model; and the transactional model. The diathesis-stress model states that the interaction between stress and certain personality vulnerabilities increases risk for depression, with females being more likely than males to experience stressors [144], and have depressogenic personality diatheses [145]. Under the mediation model, different ways of coping with stressors trigger depression, and males and females differ in their tendencies to use more or less depressogenic coping styles [146]. The transactional approach emphasizes the cyclical nature of depression and proposes that individuals with depression engage in behaviors that perpetuate symptoms [147,148], with individuals with depression, more of whom are female, more likely to engage in such behaviors. 

The emergence of the gender difference in depression in adolescence is likely overdetermined: there is empirical support for many of the variables, as well as all of the models that have been proposed [86]. Given this, it seems reasonable to assume that the gender difference in depression is overdetermined throughout the life span. Thus, in order to understand the lifespan development of the gender difference in depression, we recommend research approaches that combine or compare multiple models (e.g., [90]). We also recommend the examination of variables and hypotheses that predict the emergence of the gender difference in adolescence and explain the gender difference in depression in young adults. Only by doing this, will it be possible to determine whether the gender difference in depression in the elderly is predicted by the same factors that contribute to the gender difference at younger ages. This may reveal important developmental processes that are involved in the onset and perpetuation of gender differences in depression.

### 7.3. Meta-Analytic Research

We strongly recommend that a comprehensive meta-analytic review of the empirical literature on the gender difference in depression, including the empirical literature on the gender difference in older adults, be conducted as the next step towards understanding gender and depression over the lifespan. Some meta-analyses have already been conducted on subsets of older adults (e.g., [108]). However, a large-scale meta-analysis that includes younger and older samples would reveal whether the magnitude of the gender difference in depression changes over the life span. In the present review, we found mixed evidence for the shape of the gender difference over the life span, with studies being nearly evenly split between findings that the gender difference narrows in older cohorts, widens in older cohorts, or stays the same across age groups. Only a meta-analytic review would be able to quantify the effect size of the gender difference in depression across multiple studies of the lifetime prevalence of depression. We recommend that the predictors of the gender difference in depression that are discussed in the present review be examined as potential moderators of the gender difference in older adults. 

### 7.4. Gaps in the Literature

A critical issue that needs to be addressed in future research on gender, depression, and aging, is stratification by age group in individuals over the age of 60. Most studies included in the present review lumped all of the individuals over the age of 60 together in their analyses. This can be contrasted with research on adolescents and young adults where narrow cross-sectional cohorts are carefully defined. In addition, given the expected life span, at least in developed countries, individuals at age 60 can expect to live for approximately 20 more years. Thus, research that combines together participants over age 60 would be similar to research that combines individuals between the ages of 20 and 40. There are important changes in health, ability, and marital status that take place in the decades following age 60. Ideally, research on gender differences and depression in older adults should stratify findings by age in order to examine the correlates of developmental changes that are particular to different decades. Future studies on gender differences in late life depression should stratify by age, examine interactions between gender and age, and identify predictors that particularly affect individuals in different age categories.

The studies in the empirical literature that examined the gender difference in depression across large parts of the life span used cross-sectional designs [72,73,75]. Such designs can confound inter-individual with inter-cohort differences. Thus, we recommend that studies adopting this approach should take care to isolate factors that predict intercohort differences. Seedat and colleagues (2009) found that the gender difference in depression has narrowed in more recent cohorts, and that changes in traditional gender roles may account for this difference [75]. However, others have found the opposite effect, where the gender difference in depression is smaller in older cohorts than in more recent cohorts of younger adults [72,76]. Cohort-level changes in mental health awareness and stigma, the acceptance of negative affectivity, and developmental changes in coping and sociomemotional selectivity have all been proposed to account for this pattern [9,72,149]. Ideally, studies that stratify by cohort should examine multiple factors that represent possible intercohort differences, and at the same time, could contribute variance to the gender difference over time. 

Some of the studies included in this review followed multiple cohorts longitudinally for a number of years, but none of them followed the cohorts long enough to collect life span developmental data (e.g., [80,111]). It is obviously extremely difficult to follow a single cohort across the life span. So, it is not surprising that studies reporting on the gender difference in depression have generally not tried to use this approach. It would be useful to have studies that combine a cross-sectional with a longitudinal approach. For example, a study that began with cohorts sampled every 10 years across the life span, and then continued with yearly follow ups over the subsequent 10 years, would make it possible to separate inter-individual from inter-cohort differences.

Using self-report data with elderly participants, several studies have found that the gender difference in first-onset of depression follows an inverted U-shaped function across the lifespan, with the gender difference converging in adolescence and around age 65, and widening from adolescence to middle adulthood [83,84,150]. Zisook and colleagues’ (2004) data show that women are more likely than men to experience early initial episodes of depression, which are linked to more severe and enduring depression [150]. Longitudinal research would be useful in identifying the trajectory of depression after first onset, and whether the gender difference in the early onset of depression accounts for some of the variance in the gender difference in depression at least until age 65.

The majority of studies included in the present review did not report on screening for comorbid psychological disorders, dementia, or health problems, and those that did screen for these issues usually did not exclude participants based on that screening. As a result, it is difficult to determine whether the reported gender difference in depression is due, at least in part, to other health-related factors. Older adults may present with depression as a symptom or as a consequence of an underlying health problem or cognitive decline [151,152,153]. We recommend that future studies use methods that provide better control of health variables. 

### 7.5. Relevance to Clinical Practice

The higher prevalence of depression in women than in men highlights the need for mental health professionals to target women in depression screenings and interventions. Women’s health initiatives would benefit from including depression as a target for educational, prevention, and treatment interventions. Since elderly men who live alone are also at a high risk for depression, they could be targeted by primary care physicians for depression screening during medical visits or in community-based depression screening programs. 

Increasing our understanding of the predictors of the gender differences in depression can help practitioners select more appropriate interventions. With women who are ruminating and using avoidance coping skills, cognitive-behavioral interventions might be best suited to treat depression. Interpersonal psychotherapy would also be an appropriate intervention for individuals for whom social support, or the lack thereof, is impacting depressive symptoms. Since women who are married are more susceptible to depression than men who are married, couple interventions may be more appropriate for married women than married men.

### 7.6. Final Thoughts

Depression in older adults is a serious concern. The experience of depression can lead to personal distress and disability, and pose a significant impediment to successful aging. Generally speaking, successful aging is a multidimensional construct that includes psychological health, physical health, social support, and the ability to carry out the tasks of everyday life [154]. Unsurprisingly, intercorrelations exist among the determinants of well-being in the elderly. Our review finds that the gender difference in depression is present in elderly samples and is similarly overdetermined. Multiple predictors account for the gender difference in depression in the elderly in complex and sometimes contradictory ways. Elucidating the interactions between the predictors and the lifespan development of the gender difference in depression will likely contribute to the timely prevention, identification, and treatment of depression in the elderly. 

## Supplementary Materials

The following are available online at www.mdpi.com/2308-3417/2/4/35/s1, Table S1: Articles reporting the gender difference in depression in older adults.
