# Supplementary material for: The Gender Difference in Depression: Are Elderly Women at Greater Risk for Depression Than Elderly Men?
Source: Geriatrics (Basel). 2017 Nov 15;2(4):35. doi: 10.3390/geriatrics2040035 (PMC6371140; doi:10.3390/geriatrics2040035)
Supplement: Supplementary file 1 [file geriatrics-02-00035-s001.pdf]

Article	Age	Participants (N)	Instrument	Country	Gender Difference	Notes
Articles that reported that women were significantly more depressed than men						
Ahern & Hendryx, 2008 [131]	64+	2546	CES-D[1]	U.S.	w>m	Participant age was 64-66
Altun & Yazici, 2015 [20]	60+	216	GDS[2]	Turkey	w>m	
Alvarado et al., 2007 [21]	60+	7649	GDS	Argentina, Barbados, Brazil, Chile, Cuba, Mexico, Uruguay	w>m	
Ancelin et al., 2010 [22]	65+	1792 (baseline)	CES-D	France	w>m	w>m at 7 year follow-up
Anstey & Luszcz, 2002 [23]	70+	1910 (baseline)	CES-D	Australia	w>m	w>m at 2 year follow-up
Back & Lee, 2011 [135]	65+	4165	CES-D	South Korea	w>m	
Barefoot et al., 2001 [24]	60+	570	OBD[3]	Denmark	w>m	Longitudinal phases at ages 60 and 80
Barry et al., 2008 [25]	70+	754 (baseline)	CES-D	U.S.	w>m	w>m at 18 mo, 36 mo, 54 mo, 72 mo follow-up
Bookwala & Schulz, 2000 [26]	65+	283	CES-D	U.S.	w>m	
Brailean et al., 2016 [27]	M=71	3107	CES-D	The Netherlands	w>m	
Brinda et al., 2016 [28]	65+	14877	ICD-10 DCR[4]	China, India, Ghana, Mexico,	w>m	

				Russia, South Africa		
Carriere et al., 2011 [29]	65+	3191 (baseline)	CES-D	France	w>m	CES-D scores more likely to increase for women compared to men over 2 year follow-up
Chan et al., 2012a [88]	65+	2630 (baseline)	GDS, face to face interview	China	w>m	w>m men at 2 year follow-up
Chan et al., 2012b [113]	60+	4489	CES-D	Singapore	w>m	
Chen et al., 2010 [30]	65+	606	HADS[5]	Taiwan	w>m	
Choi et al., 2010 [31]	M=70	736	PHQ-9[6]	U.S.	w>m	
Cyprien et al., 2014 [32]	65+	467 (baseline)	CES-D	France	w>m	Gender difference in depression not assessed at 10 year follow-up
Ermer & Proulx, 2016 [33]	67+	1009	CES-D	U.S.	w>m	
Gerst et al., 2010 [34]	75+	1699	CES-D	U.S.	w>m	
Glaesmer et al., 2011 [132]	60+	1659	PHQ-9	Germany	w>m	
Glei et al., 2013 [111]	60+	4049 (baseline)	CES-D	Taiwan	w>m	w>m at 18 year follow-up
Guo, Chi, & Silverstein, 2017 [35]	M=72	1327	CES-D	China	w>m	
Harwood et al., 1999 [36]	60+	506	HAM-D[7]	U.S.	w>m	
Hinton et al., 2006 [37]	60+	1613	SCL-20[8]	U.S.	w>m	
Hybels, Blazer, & Pieper, 2001 [38]	65+	4162	CES-D	U.S.	w>m	
Imai et al., 2015 [39]	65+	747	GDS	Japan	w>m	

Jansson et al., 2004 [40]	M=72	1918	CES-D	Sweden	w>m	Participants were 959 twin pairs
Jeon et al., 2007 [134]	65+	930	Single item depression question	South Korea	w>m	
Katsumata et al., 2005 [89]	65+	660	GDS	Japan	w>m	
Kim et al., 2007 [16]	65+	732 (baseline)	GMS[9]	South Korea	w>m	Gender difference in depression not assessed at 2.5 year follow-up
Kim et al., 2013 [130]	65+	2614	CES-D	U.S.	w>m	
Krause et al., 1995 [117]	60+	9923	CES-D	China, Taiwan, Japan, U.S.	w>m	
Leach et al., 2008 [74]	60+	2551	GADS[10]	Denmark	w>m	
Lee & Lee, 2011 [112]	65+	4155	CES-D	South Korea	w>m	
Li et al., 2015 [41]	60+	3824	CES-D	China	w>m	
Lin & Wang, 2011 [42]	65+	192	CES-D	Taiwan	w>m	
Linn, Hunter, & Harris, 1980 [43]	65+	188	HSCL[11]	U.S.	w>m	
Lohman, Dumenci, & Mezuk, 2014 [44]	65+	3665	CES-D	U.S.	w>m	
Luthy et al., 2015 [45]	65+	2888	EQ-5D[12]	Switzerland	w>m	
Lutzky & Knight, 1994 [96]	M=70	92	CES-D	U.S.	w>m	
Mair, 2010 [104]	60+	10441	CES-D	U.S.	w>m	
Nolen-Hoeksema & Aldao, 2011 [72]	65+	297	BDI[13], SCID[14]	U.S.	w>m	
Norton et al., 2006 [46]	65+	4468	DIS[15]	U.S.	w>m	
Oh et al., 2015 [116]	60+	60305	CES-D	South Korea	w>m	

Oltman, Michals, & Steer, 1980 [47]	M=70	502	WSADI[16]	U.S.	w>m	
Osborn et al., 2002 [48]	75+	14545	GDS	UK	w>m	
Palsson, Ostling, & Skoog, 2001 [85]	70+	392 (baseline)	Clinician interview using DSM-IIIR criteria	Sweden	w>m	w>m over 15 year follow-up period with 5 assessments
Park et al., 2013 [103]	60+	674	GDS	U.S.	w>m	
Park, Unutzer & Grembowski, 2014 [115]	65+	395	WMH-CIDI[17]	U.S.	w>m	
Park et al., 2012 [49]	65+	6018	GDS	South Korea	w>m	
Payne et al., 2014 [50]	62+	2261	NDSM[18]	U.S.	w>m	
Poysti et al., 2012 [136]	M=78	335	GDS	Finland	w>m	
Ried & Planas, 2002 [51]	65+	2567	CES-D	U.S.	w>m	
Schoevers et al., 2000 [139]	65+	4051 (baseline)	GMS	The Netherlands	w>m	Depression was not assessed at 6 year follow-up
Seematter-Bagnoud et al., 2010 [87]	65+	1309	Geriatric Adverse Life Events Scale[19]	France	w>m	
Sicotte et al., 2008 [52]	60+	1905	GDS	Cuba	w>m	
Sikorski et al., 2014 [53]	75+	1193	GDS	Germany	w>m	
Taheri Tanjanai, Moradinazar, & Najafi, 2017 [54]	60+	1350	GDS	Iran	w>m	

Thompson et al., 2004 [110]	M=70	61	CES-D	U.S.	w>m	
Thomsen et al., 2005 [73]	70+	302	BDI	Denmark	w>m	
Tiedt, 2010 [55]	65+	3807	CES-D	Japan	w>m	
van't Verr-Tazelaar et al., 2008 [56]	75+	2850	CES-D	The Netherlands	w>m	
Yaka et al., 2014 [57]	65+	482	GDS and clinical interview	Turkey	w>m	
Yancu, 2011 [58]	65+	2128	IAS[20]	U.S.	w>m	
Zunzunegui et al., 2015 [59]	65+	1995	CES-D	Canada, Brazil, Colombia, Albania	w>m	
Articles that reported mixed findings for the gender difference in depression						
Antonucci et al., 2002 [109]	60+	2074	CES-D	France, Germany, Japan, U.S.	w>m (except in Japan)	Separately analyzed samples from each country. In Japan, w=m.
Takkinen et al., 2004 [60]	70+	498 (baseline)	CES-D	Sweden	w>m (baseline); w=m at 4 year follow-up.	Participants were 249 (baseline) pairs of twins.
Tiedt, 2013 [107]	65+	2596 (baseline, Japan); 780 (baseline, U.S.)	CES-D	Japan, U.S.	w>m at baseline, n.s. difference at follow-up (Japan); w>m at baseline and	Participants in Japan and the U.S. were assessed at 2 year follow-up

					follow-up (U.S.)
Zunzunegui et al., 2007 [13]	75+	4449	GDS and CES-D	Israel, Italy, Netherlands, Spain, Sweden	w>m (except in Sweden)
Articles that reported a nonsignificant gender difference in depression					
Canoui-Poitrine et al., 2016 [61]	70+	1092	Clinician diagnosis with semi-structured interview	France	w=m
Forlani et al., 2014 [62]	74+	359	Clinician diagnosis based on CAMDEX-R[21] and ICD-10 criteria	Italy	w=m
Forsell et al., 1995 [18]	90+	329	Clinician diagnosis using MADRS[22]and DSM-IV criteria.	Sweden	w=m
Li, Lin, & Chen, 2011 [63]	65+	220	GDS	Taiwan	w=m
Lichtenberg et al., 1993 [64]	60+	180	GDS	U.S.	w=m
McLaren et al., 2007 [65]	M=71	351	Zung SDS[23]	Australia	w=m
Meller, Fichter, & Schroppel, 1997 [19]	85+	402	HAM-D, GMS	Germany	w=m

Neri et al., 2012 [133]	65+	176	GDS	Brazil	w=m
Nyunt et al., 2009 [66]	60+	4253	GDS	Singapore	w=m
Pachana et al., 2012 [17]	82+	189	PHQ-9	Australia	w=m
Robb, Small, & Haley, 2008 [67]	60+	238	GDS	U.S.	w=m
Russell & Taylor, 2009 [119]	60+	947	CES-D	U.S.	w=m
Sutin et al., 2010 [68]	M=71	100	CES-D	U.S.	w=m
Zhang & Li, 2011 [118]	M=72	1428	CES-D	China	w=m
Articles that reported that men were significantly more depressed than women					
Djukanovic, Sorjonen, & Peterson, 2015 [69]	65+	6659	HADS	Sweden	m>w
Lim, 2014 [70]	65+	317	GDS	South Korea	m>w

Note. 1=Center for Epidemiologic Studies Depression Scale, 2=Geriatric Depression Scale, 3=Obvious Depression Scale, 4=International Statistical Classification of Disease and Related Health Problems, 10th Revision with Diagnostic Criteria for Research, 5=Hospital Anxiety and Depression Scale, 6=Patient Health Questionnaire 9, 7=Hamilton Rating Scale for Depression, 8=Symptom Checklist Depression Scale, 9=Geriatric Mental State Schedule, 10=Goldberg Anxiety and Depression Scales, 11=Hopkins Symptom Checklist, 12=EuroQoL 5-Dimensions Questionnaire, 13=Beck Depression Inventory, 14=Structured Clinical Interview for DSM-IV, 15=NIMH Diagnostic Interview Schedule, 16=Wakefield Self-Assessment Depression Inventory, 17= World Mental Health version of the World Health Organization Composite International Diagnostic Interview, 18= NSHAP Depressive Symptoms Measure, 19=Geriatric Adverse Life Events Scale, 20=Index of Affective Suffering, 21=Cambridge Mental Disorders of the Elderly Examination-Revised, 22=Montgomery Asberg Depression Rating Scale, 23=Zung Depression Scale.
